# Molecular detection of *Anaplasma* spp., *Babesia* spp. and *Theileria* spp. in yaks (*Bos grunniens*) and Tibetan sheep (*Ovis aries*) on the Qinghai-Tibetan Plateau, China

**DOI:** 10.1186/s13071-021-05109-2

**Published:** 2021-12-23

**Authors:** Yongcai He, Wangkai Chen, Ping Ma, Yaoping Wei, Ruishan Li, Zhihong Chen, Shuyu Tian, Tongsheng Qi, Jinfang Yang, Yali Sun, Jixu Li, Ming Kang, Ying Li

**Affiliations:** 1 State Key Laboratory of Plateau Ecology and Agriculture, Qinghai University, Xining, 810016 China; 2 College of Agriculture and Animal Husbandry, Qinghai University, Xining, 810016 China

**Keywords:** Tick-borne pathogens, Yak, Tibetan sheep, Qinghai-Tibet Plateau

## Abstract

**Background:**

*Anaplasma*, *Babesia* and *Theileria* are tick-borne pathogens (TBPs) that affect livestock worldwide. However, information on these pathogens in yaks (*Bos grunniens*) and Tibetan sheep (*Ovis aries*) on the Qinghai-Tibet Plateau (QTP), China, is limited. In this study, *Anaplasma* spp., *Babesia* spp. and *Theileria* spp. infections were assessed in yaks and Tibetan sheep from Qinghai Province.

**Methods:**

A total of 734 blood samples were collected from 425 yaks and 309 Tibetan sheep at nine sampling sites. Standard or nested polymerase chain reaction was employed to screen all the blood samples using species- or genus-specific primers.

**Results:**

The results showed that 14.1% (60/425) of yaks and 79.9% (247/309) of Tibetan sheep were infected with at least one pathogen. *Anaplasma ovis*, *Anaplasma bovis*, *Anaplasma capra*, *Anaplasma phagocytophilum*, *Babesia bovis* and *Theileria* spp*.* were detected in this study, with total infection rates for all the assessed animals of 22.1% (162/734), 16.3% (120/734), 23.6% (173/734), 8.2% (60/734), 2.7% (20/734) and 19.3% (142/734), respectively. For yaks, the infection rate of *A. bovis* was 6.4% (27/425), that of *B. bovis* was 4.7% (20/425) and that of *Theileria* spp. was 3.3% (14/425). Moreover, 52.4% (162/309) of the Tibetan sheep samples were infected with *A. ovis*, 30.1% (93/309) with *A. bovis*, 56.0% (173/309) with *A. capra*, 19.4% (60/309) with *A. phagocytophilum* and 41.4% (128/309) with *Theileria* spp.

**Conclusions:**

This study revealed the prevalence of *Anaplasma* spp., *Babesia* spp. and *Theileria* spp. in yaks and Tibetan sheep in Qinghai Province, China, and provides new data for a better understanding of the epidemiology of TBPs in these animals in this area of the QTP, China.

**Graphical Abstract:**

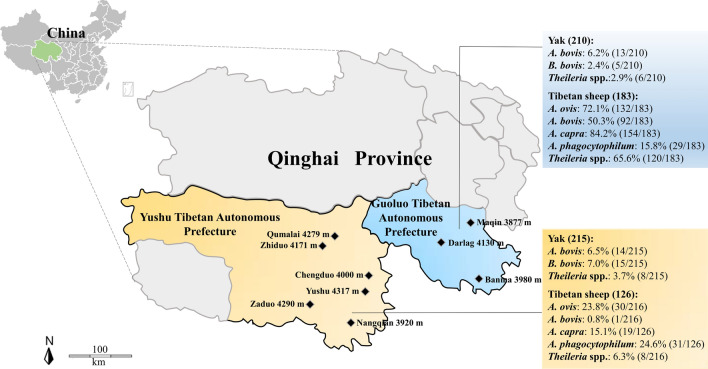

**Supplementary Information:**

The online version contains supplementary material available at 10.1186/s13071-021-05109-2.

## Background

The Qinghai-Tibet Plateau (QTP), the largest and highest plateau in the world (and thus sometimes referred to as the “roof of the world” or the “third pole” [1, 2]), is located in northwest China. A variety of domestic livestock are maintained on the QTP, including yaks (*Bos grunniens*), Tibetan sheep (*Ovis aries*), cattle, Mongolian sheep, goats, camels (*Camelus bactrianus*) and horses [3]. Yak and Tibetan sheep are indigenous to the QTP, and provide local herdsmen with milk, meat, fuel (yak dung) and wool [4].

In recent years, tick-borne pathogens (TBPs) have attracted increasing attention due to the economic losses they cause in animal production and the risk they pose to humans. Tibetan sheep and yaks range freely on the high-altitude QTP, a harsh environment with a cold climate and low rainfall, and share pastureland with other animals there [5]. This increases the potential risk of transmission of pathogens, including TBPs, such as *Anaplasma*, *Babesia* and *Theileria*, the respective etiological agents of anaplasmosis, babesiosis and theileriosis in animals [6].

*Anaplasma*, a genus of the class Alphaproteobacteria, are Gram-negative obligate intracellular pathogens which are transmitted by hard ticks to vertebrate hosts and infect different blood cells of the host [7]. In sheep and cattle, infection with these bacteria is characterized by high fever and fatigue, loss of appetite, a sudden decrease in milk production, miscarriage, stillbirth, low fertility, decreased semen quality and other clinical symptoms [8]. *Anaplasma* infections have been reported in many areas of China. For example, *Anaplasma ovis*, *Anaplasma bovis* and *Anaplasma phagocytophilum* have been detected in sheep in Qinghai Province and the Xinjiang Uygur Autonomous Region [9–13], while *Anaplasma capra*, *Anaplasma marginale*, *Anaplasma centrale* and *Anaplasma platys* have been detected in both humans and cattle in Heilongjiang Province and Chongqing City, China [6, 14].

The genus *Babesia* was discovered from the red blood cells of cattle in Romania in the nineteenth century [15]. Bovine babesiosis is caused by *Babesia bigemina*, *Babesia bovis*, *Babesia divergens*, *Babesia major* and *Babesia occultans*. In an acute case of bovine babesiosis, the main clinical features include high fever, loss of appetite, anemia, hemoglobinuria and lethargy [16], and the disease in farm animals leads to economic losses for farmers. *Babesia* spp., including *Babesia motasi-*like,* Babesia* sp. BQ1 (Lintan and Ningxian), *Babesia* sp. Tianzhu and *Babesia* sp. Hebei subgroups, have been found in and identified from sheep and goats in 16 provinces of China [17]*.* Investigations have also been undertaken on *B. bovis*, *B. bigemina* and *B. ovata* infections in beef cattle, dairy cattle and yaks in 14 provinces in China [18].

*Theileria* is an obligate intracellular hemoprotozoan parasite which is transmitted by ixodid ticks and affects a range of domestic and wild animals. Theileriosis leads to a decline in the growth rate and productivity of infected animals, and thus is a limiting factor in the development of animal husbandry [19]. On the eastern Tibetan Plateau in China (Sichuan Province), infections with *Theileria sinensis*, *Theileria luwenshuni* and *Theileria equi* have been detected in yaks, Tibetan sheep and Tibetan horses [20]. Moreover, *Theileria orientalis* [21], *Theileria uilenbergi* [13], *Theileria ovis* and *Theileria* spp. [22] have also been identified in cattle and yaks in northwestern China.

Epidemiological and molecular information on TBP infections in livestock on the QTP is limited. The data provided herein increase the available knowledge on the epidemiology of TBPs in livestock on the QTP, and provide a theoretical basis for the prevention and treatment of these pathogens in this area of China.

## Methods

### Blood sample collection and DNA extraction

**Fig. 1:**
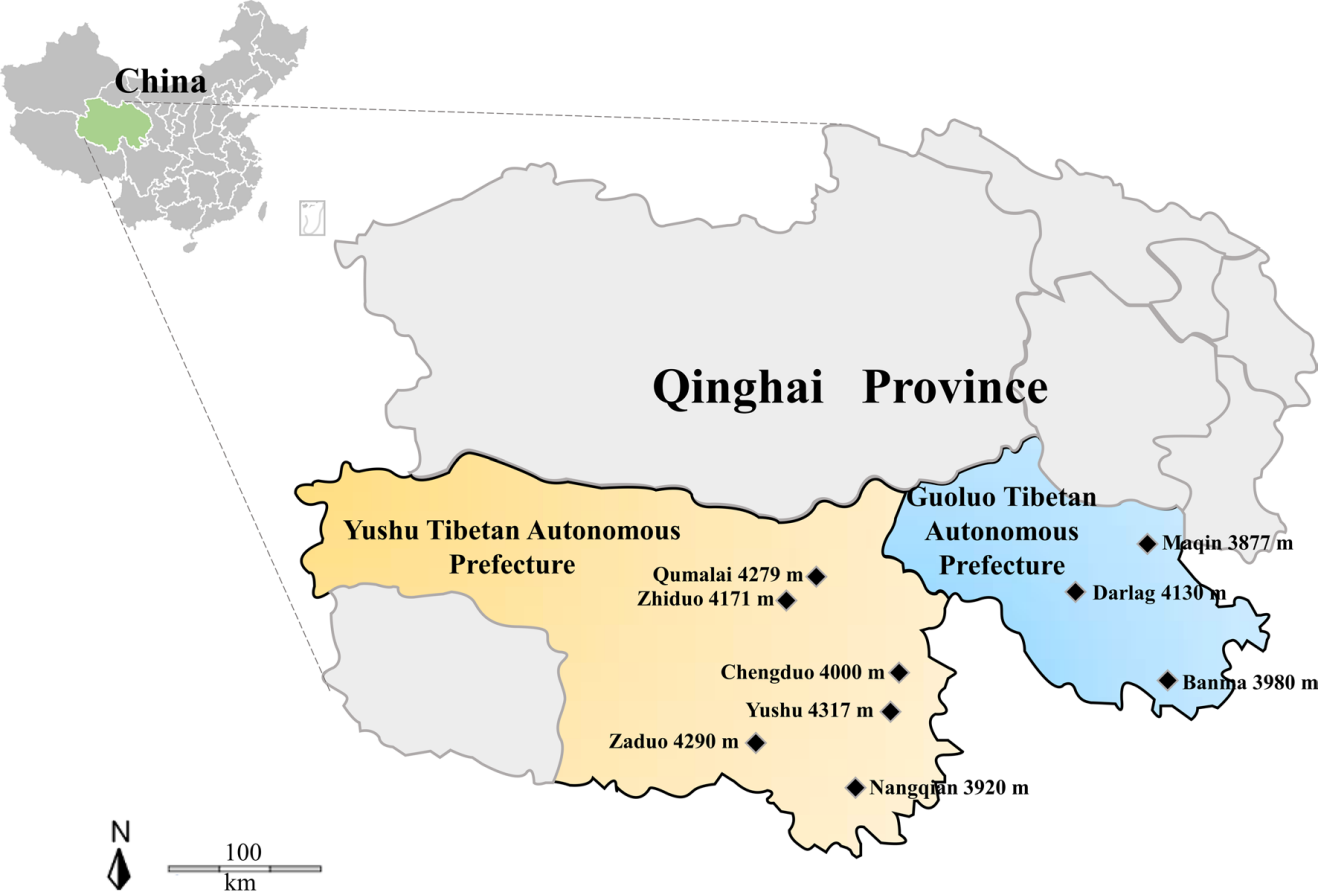
Map of the Qinghai-Tibetan Plateau and Qinghai Province showing the sampling sites and altitude (in meters).* Rhombuses* indicate the sampling locations

A total of 734 whole blood samples (comprising those from 425 yaks and 309 Tibetan sheep) were randomly collected from animals on different farms in Guoluo Tibetan Autonomous Prefecture (hereafter ‘Guoluo’) and Yushu Tibetan Autonomous Prefecture (hereafter ‘Yushu’) in the Sanjiangyuan area (which is sometimes referred to as the “water tower of China”) of Qinghai Province (Fig. 1; Additional file 1: Table S1). Blood samples were taken from the jugular vein and collected in tubes containing ethylenediaminetetraacetic acid. Genomic DNA was extracted using the MagPure Blood DNA KF Kit (Magen, China) according to the manufacturer’s manual. The DNA concentration was confirmed using a K5800 ultramicro spectrophotometer (Kaiao Technology, China), and the DNA was stored at − 80 °C until further use.

### Pathogen detection by polymerase chain reaction

Standard or nested polymerase chain reaction (PCR) was employed to screen all blood samples using species- or genus-specific primers (Additional file 1: Table S2), including *A. ovis* major surface protein 4 (*msp4*) [23], *A. bovis* 16S ribosomal RNA (16S rRNA) [24], *A. capra* citrate synthase (*gltA*) [25], *A. phagocytophilum* 16S rRNA [26], *A. marginale msp4* [23], *Babesia ovis* 18S ribosomal RNA (18S rRNA) [27], *B. bovis* spherical body protein 4 (*SBP4*) [28], *B. bigemina* rhoptry-associated protein 1a (*rap1a*) [28], *B. motasi*-like Lintan/Ningxian/Tianzhu rhoptry-associated protein 1b (*rap1b*) [17], and *Theileria* spp. 18S rRNA [29]. The PCR mixture consisted of 2 µl of DNA template, 0.5 µl each of forward and reverse primer (100 μM), 0.1 µl of *Taq* polymerase (0.5 U; New England BioLabs, USA), 0.2 µl of deoxyribonucleotide triphosphate (200 μM; New England BioLabs, USA), 1 µl of 10× ThermoPol Reaction Buffer (New England BioLabs), and double-distilled water for a total volume of 10 µl. DNA samples from the blood of animals infected with the respective pathogens, which had been collected and stored properly in previous studies, were used as positive controls. Double-distilled water was used as a negative control.

### Sequencing and phylogenetic analyses

The positive PCR products of 30% of each organism were selected randomly and sequenced. The PCR products were purified using the EasyPure Quick Gel Extraction Kit (TransGen, China) and cloned into the pMD19 T vector, which was transformed into competent *Escherichia coli* DH5α cells using the pMD19 (Simple) T-Vector Cloning Kit (TaKaRa, Japan). At least two positive clones were sequenced at Sangon Biotech (Shanghai). The nucleotide sequence identities were determined by performing GenBank Basic Local Alignment Search Tool nucleotide (BLASTn) analysis (https://blast.ncbi.nlm.nih.gov/Blast.cgi). Phylogenetic trees based on the obtained sequences were constructed using MEGA 7.0 [30].

### Statistical analysis

The chi-square test was performed to evaluate the difference in prevalence between different parameters. Exposure variables included area (Guoluo and Yushu) and altitude (3000–4000 m and 4000–5000 m). Observed differences were considered to be statistically significant when *P* < 0.05.

## Results

### Infection rates of *Anaplasma*, *Babesia* and *Theileria* in yaks and Tibetan sheep

A total of 734 whole blood samples were collected and screened. The pathogens detected in these animals were *A. capra* (23.6%, *n* = 173), followed by *A. ovis* (22.1%, *n* = 162), *Theileria* spp. (19.3%, *n* = 142), *A. bovis* (16.3%, *n* = 120), *A. phagocytophilum* (8.2%, *n* = 60) and *B. bovis* (2.7%, *n* = 20) (Table 1). All 425 blood samples of yaks were negative for *A. ovis*, *A. capra* and *A. phagocytophilum*, and 309 blood samples of Tibetan sheep were negative for *B. bovis*. A total of 14.1% (60/425) of the yaks and 79.9% (247/309) of the Tibetan sheep were positive for at least one pathogen. In addition, 51.4% (202/393) of the animals from Guoluo and 30.8% (105/341) of the animals from Yushu were positive for at least one pathogen (Table 2).

**Table 1:** The prevalence of tick-borne pathogens (TBPs) in yaks and Tibetan sheep on the Qinghai-Tibetan Plateau (QTP)

TBPs	Prevalence (%) of TBPs								
	Yaks			Tibetan sheep			Total		
	Guoluo	Yushu	Total	Guoluo	Yushu	Total	Guoluo	Yushu	Total
	(*n* = 210)	(*n* = 215)	(*n* = 425)	(*n* = 183)	(*n* = 126)	(*n* = 309)	(*n* = 393)	(*n* = 341)	(*n* = 734)
*Anaplasma ovis*	0	0	0	132 (72.1)	30 (23.8)	162 (52.4)	132 (33.6)	30 (8.8)	162 (22.1)
*Anaplasma bovis*	13 (6.2)	14 (6.5)	27 (6.4)	92 (50.3)	1 (0.8)	93 (30.1)	105 (26.7)	15 (4.4)	120 (16.3)
*Anaplasma capra*	0	0	0	154 (84.2)	19 (15.1)	173 (56.0)	154 (39.2)	19 (5.6)	173 (23.6)
*Anaplasma phagocytophilum*	0	0	0	29 (15.8)	31 (24.6)	60 (19.4)	29 (7.4)	31 (9.1)	60 (8.2)
*Babesia bovis*	5 (2.4)	15 (7.0)	20 (4.7)	0	0	0	5 (1.3)	15 (4.4)	20 (2.7)
*Theileria* spp.	6 (2.9)	8 (3.7)	14 (3.3)	120 (65.6)	8 (6.3)	128 (41.4)	126 (32.1)	16 (4.7)	142 (19.3)

**Table 2:** Single and mixed infections of TBPs in yaks and Tibetan sheep on the QTP

TBPs	Prevalence (%) of TBPs								
	Yaks			Tibetan sheep			Total		
	Guoluo	Yushu	Total	Guoluo	Yushu	Total	Guoluo	Yushu	Total
	(*n* = 210)	(*n* = 215)	(*n* = 425)	(*n* = 183)	(*n* = 126)	(*n* = 309)	(*n* = 393)	(*n* = 341)	(*n* = 734)
*Anaplasma ovis* (*A. o.*)	0	0	0	1 (0.5)	13 (10.3)	14 (4.5)	1 (0.3)	13 (3.8)	14 (1.9)
*Anaplasma bovis* (*A. b*)	13 (6.2)	14 (6.5)	27 (6.4)	3 (1.6)	0	3 (1.0)	16 (4.1)	14 (4.1)	30 (4.1)
*Anaplasma capra* (*A. c.*)	0	0	0	7 (3.8)	7 (5.6)	14 (4.5)	7 (1.8)	7 (2.1)	14 (1.9)
*Anaplasma phagocytophilum* (*A. p.*)	0	0	0	1 (0.5)	28 (22.2)	29 (9.4)	1 (0.3)	28 (8.2)	29 (4.0)
*Babesia bovis* (*B. bo.*)	5 (2.4)	14 (6.5)	19 (4.5)	0	0	0	5 (1.3)	14 (4.1)	19 (2.6)
*Theileria* spp. (*T.*)	6 (2.9)	7 (3.3)	13 (3.1)	5 (2.7)	2 (1.6)	7 (2.3)	11 (2.8)	9 (2.6)	20 (2.7)
*A. o.* + *A. b.*	0	0	0	4 (2.2)	0	4 (1.3)	4 (1.0)	0	4 (0.5)
*A. o.* + *A. c.*	0	0	0	13 (7.1)	10 (7.9)	23 (7.4)	13 (3.3)	10 (2.9)	23 (3.1)
*A. o.* + *A. p.*	0	0	0	0	2 (1.6)	2 (0.6)	0	2 (0.6)	2 (0.3)
*A. o.* + *T.*	0	0	0	0	4 (3.2)	4 (1.3)	0	4 (1.2)	4 (0.5)
*A. b.* + *A. c.*	0	0	0	2 (1.1)	0	2 (0.6)	2 (0.5)	0	2 (0.3)
*A. b.* + *A. p.*	0	0	0	0	1 (0.8)	1 (0.3)	0	1 (0.3)	1 (0.1)
*A. c.* + *A. p.*	0	0	0	2 (1.1)	0	2 (0.6)	2 (0.5)	0	2 (0.3)
*A. c.* + *T.*	0	0	0	13 (7.1)	1 (0.8)	14 (4.5)	13 (3.3)	1 (0.3)	14 (1.9)
*A. p.* + *T.*	0	0	0	1 (0.5)	0	1 (0.3)	1 (0.3)	0	1 (0.1)
*B. bo.* + *T.*	0	1 (0.5)	1 (0.2)	0	0	0	0	1 (0.3)	1 (0.1)
*A. o.* + *A. b.* + *A. c.*	0	0	0	17 (9.3)	0	17 (5.5)	17 (4.3)	0	17 (2.3)
*A. o.* + *A. b.* + *T.*	0	0	0	5 (2.7)	0	5 (1.6)	5 (1.3)	0	5 (0.7)
*A. o.* + *A. c.* + *A. p.*	0	0	0	6 (3.3)	0	6 (1.9)	6 (1.5)	0	6 (0.8)
*A. o.* + *A. c.* + *T.*	0	0	0	22 (12.0)	1 (0.8)	23 (7.4)	22 (5.6)	1 (0.3)	23 (3.1)
*A. o.* + *A. p.* + *T.*	0	0	0	3 (1.6)	0	3 (1.0)	3 (0.8)	0	3 (0.4)
*A. b.* + *A. c.* + *T.*	0	0	0	11 (6.0)	0	11 (3.6)	11 (2.8)	0	11 (1.5)
*A. o.* + *A. b.* + *A. c.* + *A. p.*	0	0	0	3 (1.6)	0	3 (1.0)	3 (0.8)	0	3 (0.4)
*A. o.* + *A. b.* + *A. c.* + *T.*	0	0	0	46 (25.1)	0	46 (14.9)	46 (11.7)	0	46 (6.3)
*A. o.* + *A. c.* + *A. p.* + *T.*	0	0	0	11 (6.0)	0	11 (3.6)	11 (2.8)	0	11 (1.5)
*A. o.* + *A. b.* + *A. c.* + *A. p.* + *T.*	0	0	0	2 (1.1)	0	2 (0.6)	2 (0.5)	0	2 (0.3)
Total mixed infections	0	1 (0.5)	1 (0.2)	161 (88.0)	19 (15.1)	180 (58.3)	161 (41.0)	20 (5.9)	181 (24.7)
Total positive	24 (11.4)	36 (16.7)	60 (14.1)	178 (97.3)	69 (54.8)	247 (79.9)	202 (51.4)	105 (30.8)	307 (41.8)

For other abbreviations, see Table 1

The infection rates of *B. bovis* in yaks were significantly higher in Yushu compared to Guoluo (*χ*^2^ = 4.56, *df* = 1, *P* = 0.0328). The infection rates of *A. ovis* (*χ*^2^ = 23.77, *df* = 1, *P* < 0.0001), *A. bovis* (*χ*^2^ = 52.14, *df* = 1, *P* < 0.0001), *A. capra* (*χ*^2^ = 46.81, *df* = 1, *P* < 0.0001) and *Theileria* spp. (*χ*^2^ = 50.75, *df* = 1, *P* < 0.0001) in Tibetan sheep were significantly higher in Guoluo compared to Yushu. In addition, the infection rates of *A. phagocytophilum* in Tibetan sheep and *B. bovis* and *Theileria* spp*.* in yaks were significantly correlated with altitude (Table 3). Infection rates of *Theileria* spp. (*χ*^2^ = 6.02, *df* = 1, *P* = 0.0141), *B. bovis* (*χ*^2^ = 5.77, *df* = 1, *P* = 0.0163) and *A. phagocytophilum* (*χ*^2^ = 23.10, *df* = 1, *P* < 0.0001) were significantly different between the groups at 3000- to 4000-m and 4000- to 5000-m altitude (Table 3).

**Table 3:** The infection rates of TBPs in yaks and Tibetan sheep by prefecture and altitude of the sampling sites

Animal	Parameter	Number of positive samples (infection rate %)					
		*Anaplasma ovis*	*Anaplasma bovis*	*Anaplasma capra*	*Anaplasma phagocytophilum*	*Babesia bovis*	*Theileria* spp.
Yak	Prefecture						
	Guoluo	0	13 (6.2)	0	0	5 (2.4)	6 (2.9)
	Yushu	0	14 (6.5)	0	0	15 (7.0)	8 (3.7)
	Statistical analysis						
	*χ*^2^		0.02			4.56	0.23
	*df*		1			1	1
	*P*-value		0.8987			0.0328	0.6293
	Altitude (m)						
	3000–4000	0	13 (5.8)	0	0	5 (2.2)	12 (5.4)
	4000–5000	0	14 (6.9)	0	0	15 (7.4)	2 (1.0)
	Statistical analysis						
	*χ*^2^		0.19			5.77	6.02
	*df*		1			1	1
	*P*-value		0.6628			0.0163	0.0141
Tibetan sheep	Prefecture						
	Guoluo	132 (72.1)	92 (50.3)	154 (84.2)	29 (15.8)	0	120 (65.6)
	Yushu	30 (23.8)	1 (0.8)	19 (15.1)	31 (24.6)	0	8 (6.3)
	Statistical analysis						
	*χ*^2^	23.77	52.14	46.81	2.44		50.75
	*df*	1	1	1	1		1
	*P*-value	< 0.0001	< 0.0001	< 0.0001	0.1185		< 0.0001
	Altitude (m)						
	3000–4000	92 (50.3)	46 (25.1)	107 (58.5)	55 (30.1)	0	80 (43.7)
	4000–5000	70 (55.6)	47 (37.3)	66 (52.4)	5 (4.0)	0	48 (38.1)
	Statistical analysis						
	*χ*^2^	0.26	2.78	0.32	23.10		0.41
	*df*	1	1	1	1		1
	*P*-value	0.6108	0.0956	0.5721	< 0.0001		0.5242

### Sequencing analysis

Table 4 shows the 38 representative sequences that were submitted to GenBank from this study.

**Table 4:** Accession numbers of DNA sequences from this study deposited in GenBank

Obtained sequences					Closest BLASTn match		
Pathogen	Animal	Target gene	Accession number	Length (bp)	Identity (%)	Pathogen isolate	Accession number (host, country)
*Anaplasma ovis*	Tibetan sheep	*msp4*	MZ130286	347	100	*A. ovis*	MN39479 (sheep, China)
	Tibetan sheep		MZ130287	347	100	*A. ovis*	MN39479 (sheep, China)
*Anaplasma bovis*	Tibetan sheep	16S rRNA	MZ069105	551	99.46	*A. bovis*	KJ659040 (sika deer, China)
	Yak		MZ069106	551	99.64	*A. bovis*	KJ659040 (sika deer, China)
	Yak		MZ069107	551	99.82	*A. bovis*	MT036513 (sheep, Russia)
	Yak		MZ069108	550	99.82	*A. bovis*	MT036513 (sheep, Russia)
	Yak		MZ069109	551	99.82	*A. bovis*	MT036513 (sheep, Russia)
	Yak		MZ069110	551	100	*A. bovis*	MT036513 (sheep, Russia)
	Yak		MZ069111	551	99.64	*A. bovis*	MT036513 (sheep, Russia)
	Yak		MZ069112	551	100	*A. bovis*	KJ639885 (red deer, China)
	Tibetan sheep		MZ069105	551	99.46	*A. bovis*	KJ659040 (sika deer, China)
*Anaplasma capra*	Tibetan sheep	*gltA*	MZ130264	793	74.43	*A. capra*	MH029895 (tick, China)
	Tibetan sheep		MZ130265	794	74.08	*A. capra*	MH029895 (tick, China)
	Tibetan sheep		MZ130266	793	74.02	*A. capra*	MH029895 (tick, China)
*Anaplasma phagocytophilum*	Tibetan sheep	16S rRNA	MZ073291	545	99.82	*A. phagocytophilum*	KC422267 (tick, North Korea)
	Tibetan sheep		MZ073292	545	99.27	*A. phagocytophilum*	MT754352 (cattle, South Korea)
	Tibetan sheep		MZ073293	545	99.63	*A. phagocytophilum*	KC422267 (tick, North Korea)
	Tibetan sheep		MZ073294	545	99.63	*A. phagocytophilum*	KC422267 (tick, North Korea)
*Babesia bovis*	Yak	*SBP4*	MZ130288	503	99.40	*B. bovis*	KX685399 (cattle, Benin)
	Yak		MZ130289	503	100	*B. bovis*	AB617641 cattle Syria
	Yak		MZ130290	503	99.80	*B. bovis*	KX685399 (cattle, Benin)
	Yak		MZ130291	503	99.80	*B. bovis*	KX685399 (cattle, Benin)
	Yak		MZ130292	503	99.80	*B. bovis*	KX685399 (cattle, Benin)
	Yak		MZ130293	503	99.60	*B. bovis*	KX685399 (cattle, Benin)
	Yak		MZ130294	503	99.80	*B. bovis*	KX685399 (cattle, Benin)
	Yak		MZ130295	503	100	*B. bovis*	KX685399 (cattle, Benin)
*Theileria* spp.	Tibetan sheep	18S rRNA	MZ047352	583	100	*T. ovis*	MN394810 (yak, China)
	Tibetan sheep		MZ047353	583	99.49	*T. ovis*	MN394810 (yak, China)
	Tibetan sheep		MZ047354	583	99.66	*T. ovis*	MN394810 (yak, China)
	Tibetan sheep		MZ047355	583	99.83	*T. ovis*	MN394810 (yak, China)
	Tibetan sheep		MZ047356	584	99.66	*T. ovis*	MN394810 (yak, China)
	Tibetan sheep		MZ047357	583	99.49	*T. ovis*	MN394810 (yak, China)
	Tibetan sheep		MZ047358	583	100	*T. ovis*	MN394810 (yak, China)
	Yak		MZ047359	583	99.83	*T. ovis*	MN394810 (yak, China)
	Yak		MZ047360	583	99.83	*T. ovis*	MN394810 (yak, China)
	Yak		MZ047361	583	99.49	*T. ovis*	MN394810 (yak, China)
	Yak		MZ047362	582	96.42	*T. ovis*	MN394810 (yak, China)
	Yak		MZ047363	583	99.14	*T. ovis*	MN394810 (yak, China)

*BLASTn* Basic Local Alignment Search Tool nucleotide,* bp* base pair,* rRNA* ribosomal RNA

The BLASTn analysis showed that the two partial sequences of the *msp4* genomic region of *A. ovis* from Tibetan sheep had 100% identity with the *A. ovis* sequence (MN39479) from sheep in China. The nine partial sequences of the *A. bovis* 16S rRNA from yaks and Tibetan sheep shared 99.1–99.8% identity with each other and 99.5–100% similarity with previously published sequences from Russia (MT036513) and China (KJ659040, KJ639885). *Anaplasma capra gltA* sequences and *A. phagocytophilum* 16S rRNA sequences from Tibetan sheep shared 99.6–99.7% and 99.1–99.8% identities with each other and 74.0–74.4% and 99.3–99.8% similarities with sequences from China (MH029895), and North and South Korea (KC422267, MT754352), respectively.

In addition, BLASTn analysis of the *SBP4* gene showed that the *B. bovis* sequences obtained in this study shared 99.0–99.8% identity with each other and 99.4–100% similarity with previously published sequences from Benin (KX685399) and Syria (AB617641). Moreover, sequence analysis revealed that the nucleotide sequences of *Theileria* spp. 18S rRNA in this study shared 96.0–100% identity with each other and 99.1–100% similarity with *T. ovis* species (MN394810) from China.

### Phylogenetic analyses

**Fig. 2:**
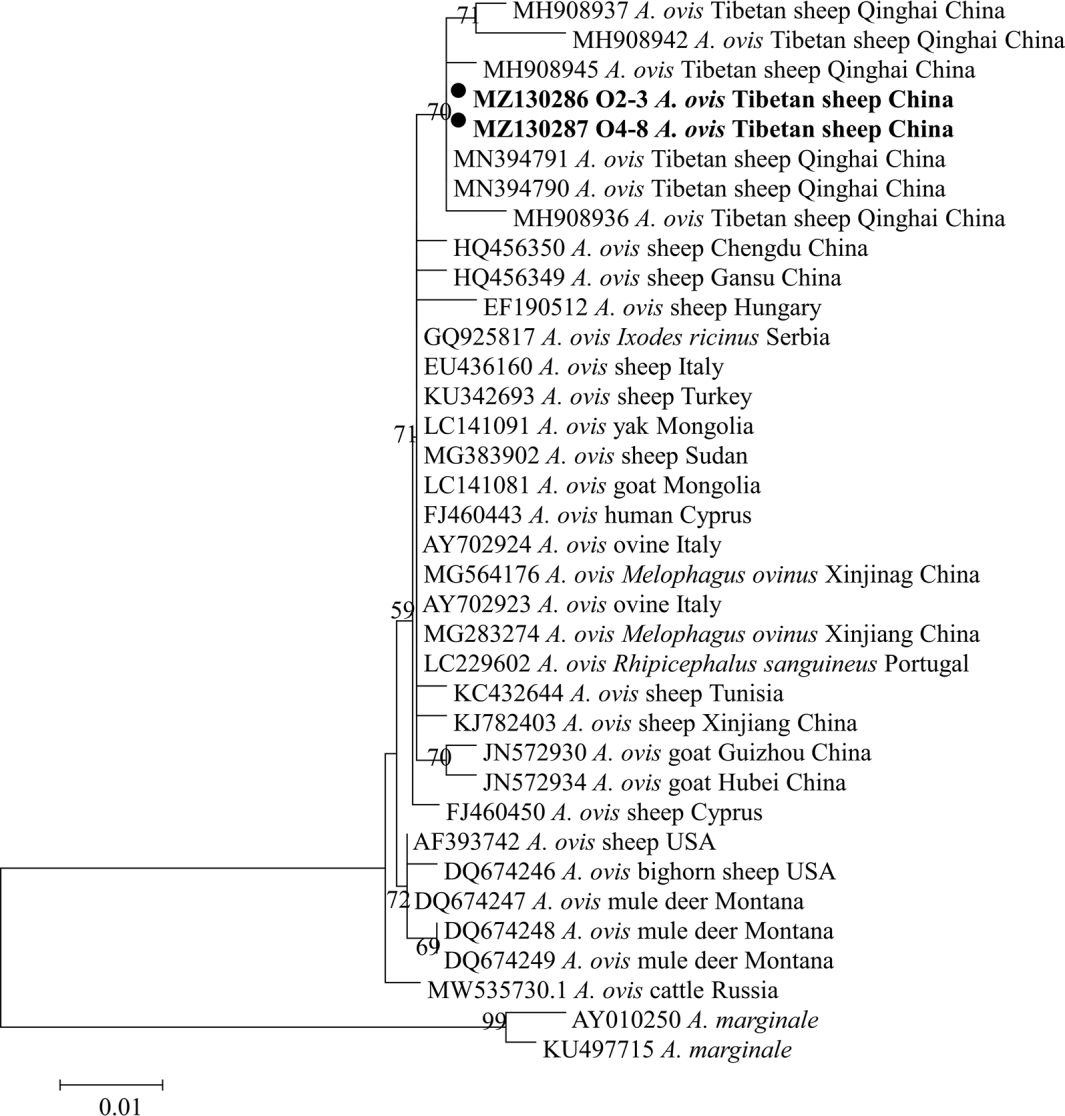
Phylogenetic tree based on *Anaplasma ovis msp4* partial sequences [347 base pairs (bp)] obtained in this study. The tree was constructed with the neighbor-joining method using MEGA7.0. Numbers at nodes represent percentage occurrence of clades in 500 bootstrap replications of data. Sequences from this study are in bold. *Anaplasma marginale* (AY010250) and *Anaplasma marginale* (KU497715) were used as outgroups. The* black circle*s indicate the sequences from Tibetan sheep in this study

**Fig. 3:**
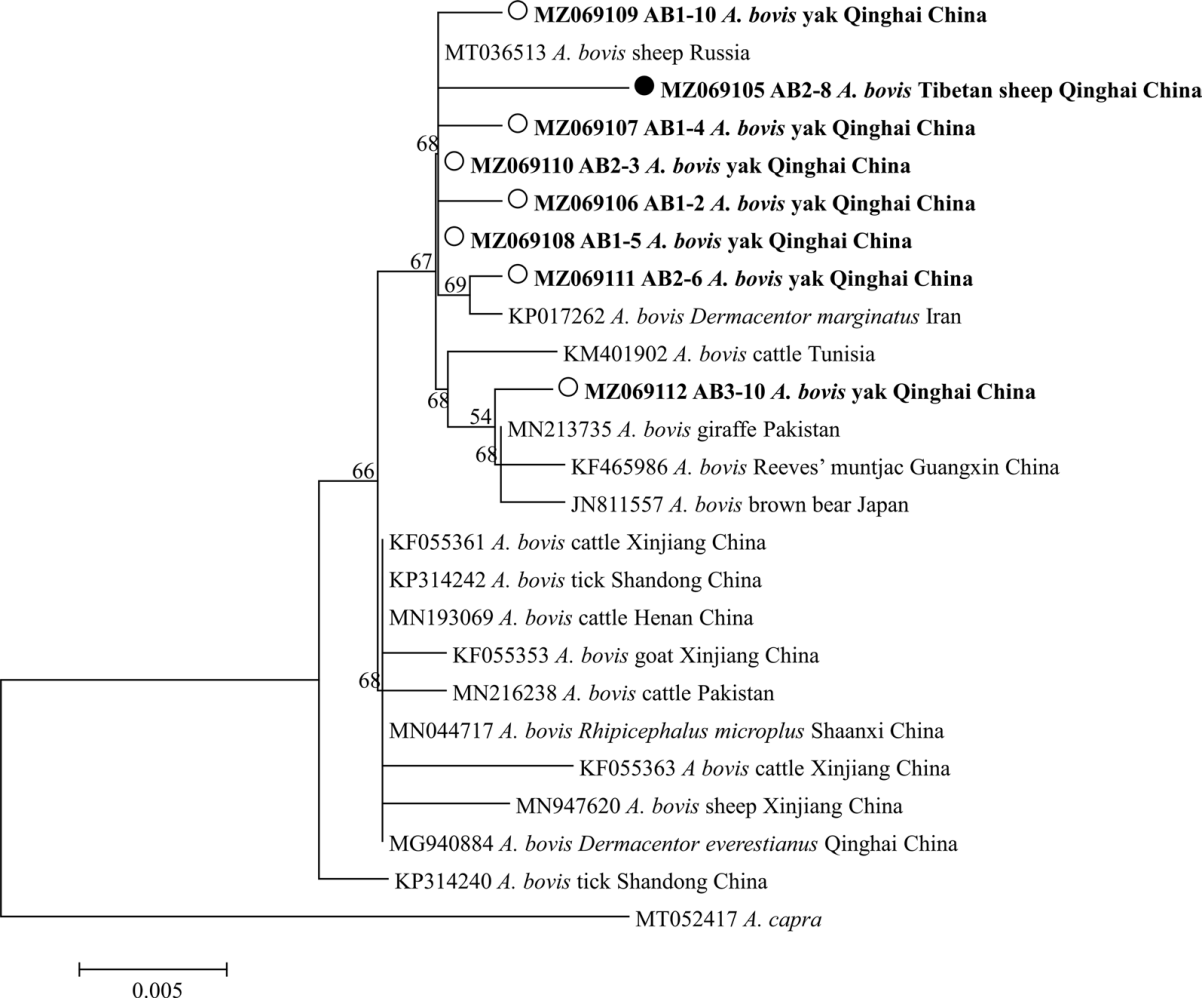
Phylogenetic tree based on *Anaplasma bovis* 16S ribosomal RNA (*rRNA*) partial sequences (551 bp) obtained in this study. The tree was constructed with the neighbor-joining method using MEGA7.0. Numbers at nodes represent percentage occurrence of clades in 500 bootstrap replications of data. Sequences from this study are in bold. *Anaplasma capra* (MT052417) was used as the outgroup. The* black circle* indicates the sequence from Tibetan sheep and the *white circles* indicate the sequences from yaks in this study

**Fig. 4:**
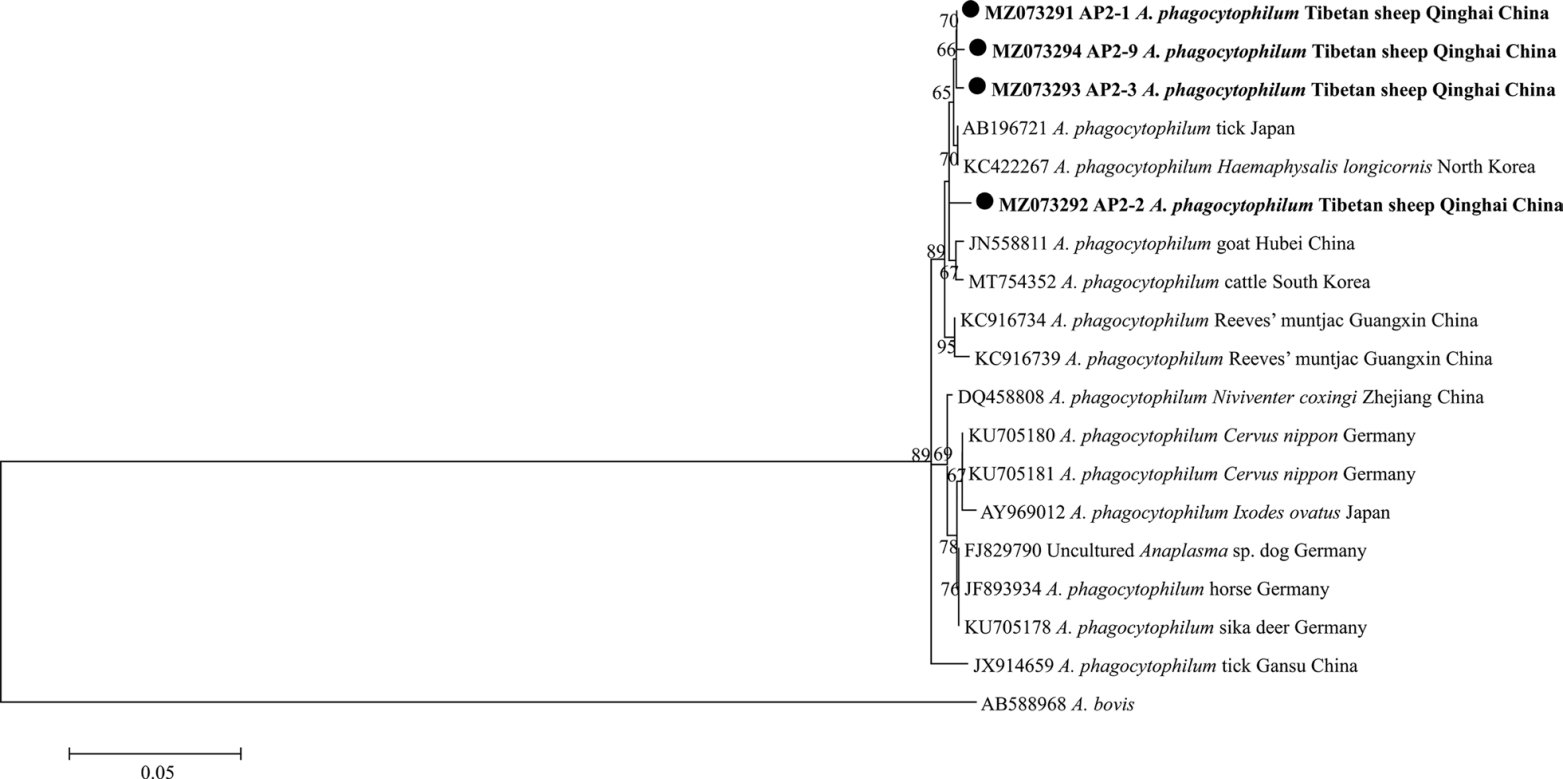
Phylogenetic tree based on *Anaplasma phagocytophilum* 16S rRNA partial sequences (546/565 bp) obtained in this study. The tree was constructed with the neighbor-joining method using MEGA7.0. Numbers at nodes represent percentage occurrence of clades in 500 bootstrap replications of data. Sequences from this study are shown in bold. *Anaplasma bovis* (AB588968) was used as the outgroup. The* black circles* indicate the sequences from Tibetan sheep in this study. For abbreviations, see Figs. 1 and 2

**Fig. 5:**
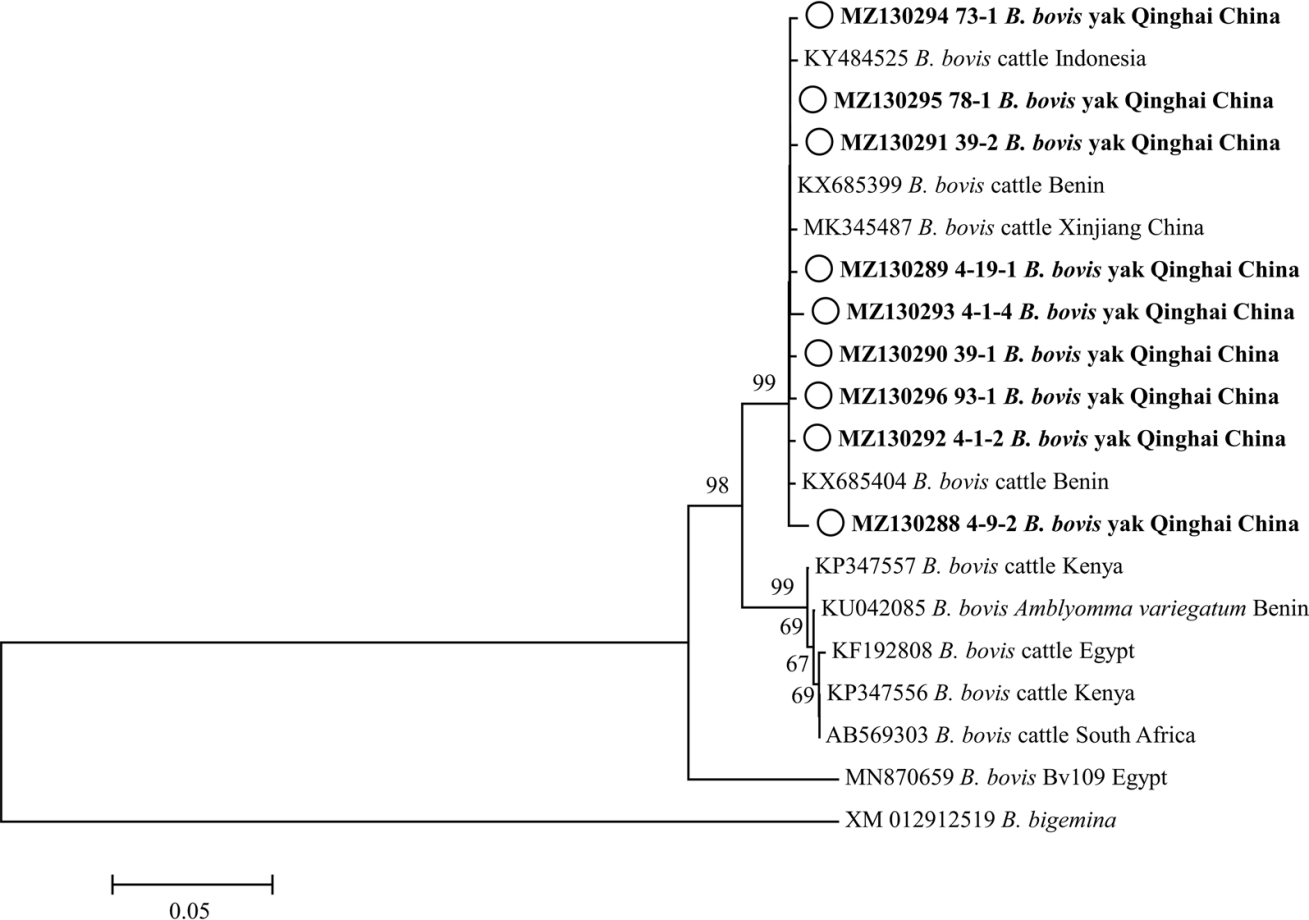
Phylogenetic tree based on *Babesia bovis msp4* partial sequences (503 bp) obtained in this study. The tree was constructed with the neighbor-joining method using MEGA7.0. Numbers at nodes represent percentage occurrence of clades in 500 bootstrap replications of data. Sequences from this study are in bold. *Babesia bigemina* (XM012912519) was used as the outgroup. The* white circles* indicate the sequences from yaks in this study

**Fig. 6:**
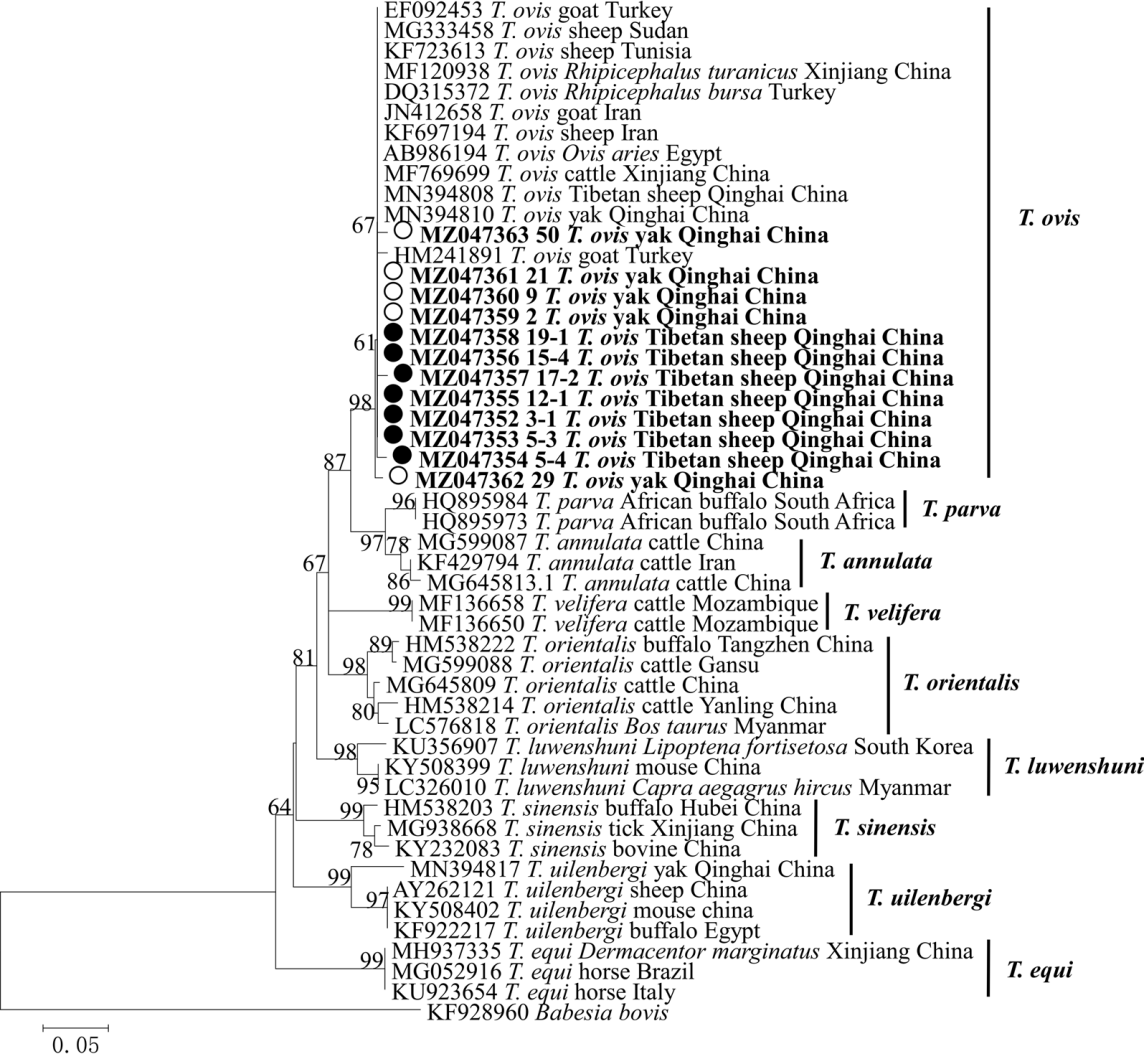
Phylogenetic tree based on *Theileria ovis* 18S rRNA partial sequences (581 bp) obtained in this study. The tree was constructed with the neighbor-joining method using MEGA7.0. Numbers at nodes represent percentage occurrence of clades in 500 bootstrap replications of data. Sequences from this study are in bold. *Babesia bovis* (KF928960) was used as the outgroup. The* black circles* indicate the sequences from Tibetan sheep and the* white circles* the sequences from yaks in this study. For abbreviations, see Figs. 1 and 2

**Fig. 7:**
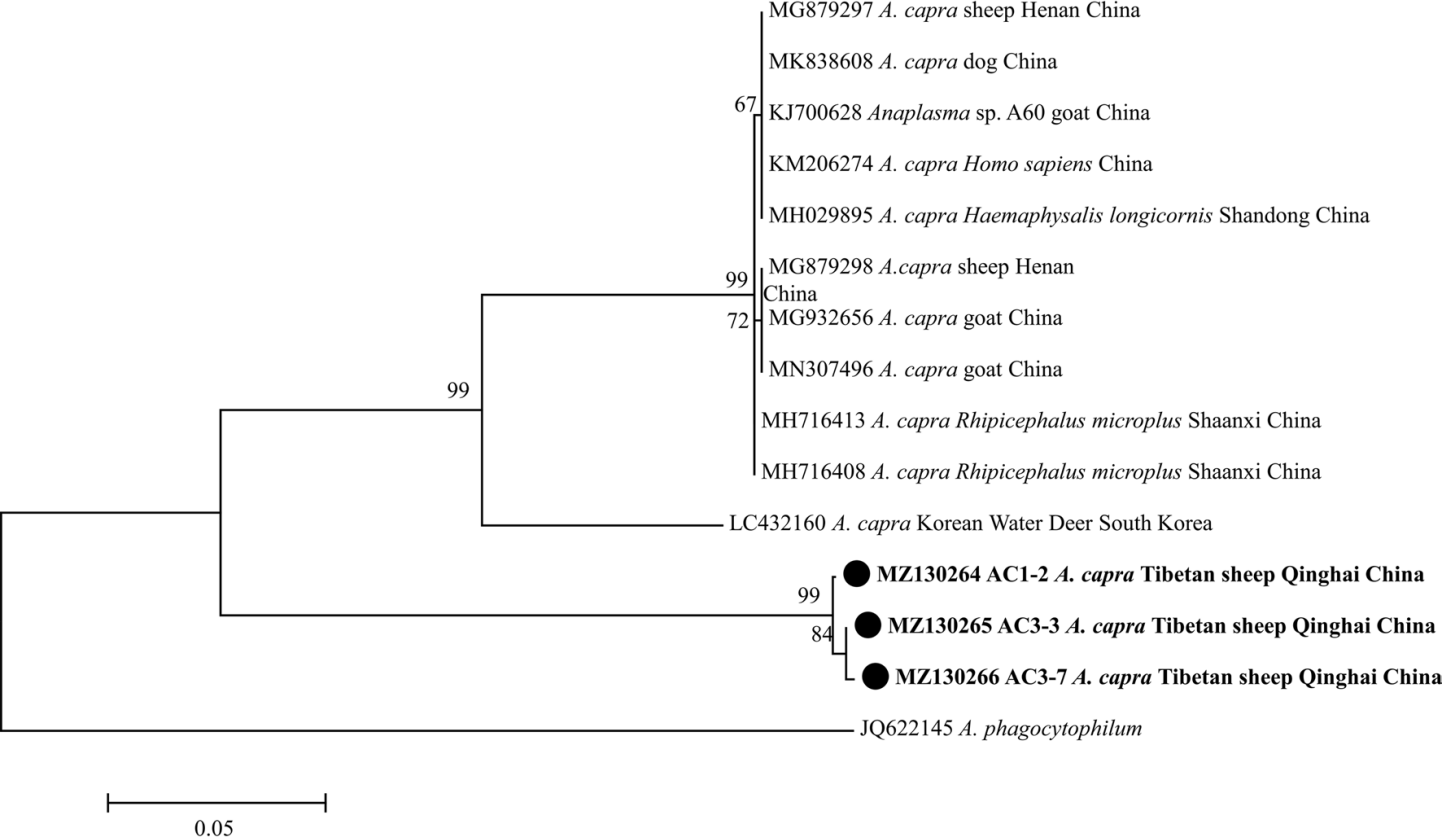
Phylogenetic tree based on *Anaplasma capra gltA* partial sequences (793 bp) obtained in this study. The tree was constructed with the neighbor-joining method using MEGA7.0. Numbers at nodes represent percentage occurrence of clades in 500 bootstrap replications of data. Sequences from this study are in bold. *Anaplasma phagocytophilum* (JQ622145) was used as the outgroup. The* black circles* indicate the sequences from Tibetan sheep in this study

Phylogenetic analysis of the sequences obtained in this study was based on the neighbor-joining method. The analysis based on the *msp4* gene of *A. ovis* in Tibetan sheep grouped the sequences from the present study into the same clade along with *A. ovis* isolates from Qinghai, China (Fig. 2). All *A. bovis* 16S rRNA sequences obtained in this study from Tibetan sheep and yaks were grouped into the same clade as isolates from Russia, Iran, Tunisia, Pakistan, Japan and China in the phylogenetic tree (Fig. 3). All four *A. phagocytophilum* 16S rRNA sequences obtained in this study were in the same clade as those from Japan, Korea and other provinces of China (Fig. 4). In the *B. bovis* phylogenetic tree, all the yak-derived sequences in this study were grouped into the same clade as those from cattle from Indonesia, Benin and China (Fig. 5). In addition, phylogenetic analysis of *Theileria* spp*.* based on the 18S rRNA gene showed that sequences obtained from the Tibetan sheep and yaks were grouped with the *T. ovis* clade with sheep, cattle, Tibetan sheep, yak and goat isolates from Iran, Egypt, China and Turkey (Fig. 6). However, in the phylogenetic tree of *A. capra*, all the obtained sequences from Tibetan sheep in the current study formed a separate branch, while other sequences of different animals from China were clustered together (Fig. 7).

## Discussion

We investigated the molecular prevalence and genetic diversity of TBPs in yaks and Tibetan sheep on the QTP to increase the amount of available epidemiological data on these pathogens in this area of China. *Anaplasma* spp., *Babesia* spp. and *Theileria* spp. were detected in the yaks and Tibetan sheep in the locations studied.

A total of four *Anaplasma* species were detected in blood samples of yaks and Tibetan sheep from Guoluo and Yushu. The infection rates of *A. ovis*, *A. capra* and *A. phagocytophilum* in Tibetan sheep were 52.4%, 56.0% and 19.4%, respectively, but none of these species were detected in yaks. *Anaplasma bovis* was detected in samples from both types of animals, although the infection rate was higher in Tibetan sheep (30.1%) than in yaks (6.4%), which suggests that the former may be more susceptible to this pathogen than the latter. *Anaplasma ovis* has not only been reported in many areas of China but also in other countries, at a positivity rate ranging from 16.05 to 83.9% [9, 10, 12, 13, 31–34]. This suggests that this pathogen, which causes sheep anaplasmosis, is an important infectious agent. *Anaplasma bovis* has also been detected in animals from different countries, such as cattle from Pakistan [35], cats from Angola [36], sheep and goats from Tunisia [37], goats from China [38] and Korean water deer from Korea [39].

 In the present study, positive rates of 19.4% and 56.0% were found for *A. phagocytophilum* and *A. capra* respectively, in Tibetan sheep. These two pathogens can infect not only ruminants but also humans [14, 40]. The infection rate of *A. phagocytophilum* in Tibetan sheep in the present study was lower than that in sheep (42.9%) and goats (38.5%) in previous studies carried out in Gansu [41]. However, the infection rate of *A. capra* was higher in the present study than in previous investigations [42]. These markedly different results may be due to the fact that *A. capra* is found in a variety of ticks, including *Haemaphysalis qinghaiensis* [42], a species of tick unique to the QTP, and grazing is more likely to increase the exposure of animals to ticks.

 The main pathogens that cause bovine babesiosis, which was first reported in China in 1948, are *B. bovis* and *B. bigemina* [43]. Previous studies on the prevalence of *B. bovis* in China found that this species was widespread in cattle in 14 provinces of the country, with infection rates ranging from 1.0 to 60.0%. Among these, the infection rate of *B. bovis* in yaks in two cities in Gansu Province, one of which is located east and the other northeast of the QTP, was 13.0%, while this species was not detected in yaks in Qinghai Province [18]. In this study, the infection rate of *B. bovis* was 4.7%, which is lower than that previously reported [18]. This could be due to differences between the studies in terms of geographic and temporal factors and vector distribution [44].

Ovine theileriosis was reported as early as 1956 in Qinghai, China [45]. This disease was originally thought to be caused by *T. ovis* [46], but infection with different *Theileria* species has been detected in different animals in China and in other countries worldwide [6, 13, 47–49]. Previous studies have reported *T. sinensis* and *T. orientalis* infections in cattle and *T. luwenshuni* and *T. uilenbergi* infections in sheep from Chongqing City and Xinjiang Province in China, respectively [6, 13]. Sequencing analysis performed in the present study showed that only *T. ovis* was present in yaks and Tibetan sheep on the QTP. The infection rates of *Theileria* in Tibetan sheep were significantly higher than those in yaks, which is a similar finding to that of a previous report [50]. The high prevalence of *T. ovis* in sheep in China and in other countries indicates that this pathogen cannot be neglected [47–54]. A study by Luo et al. [55] showed that *H. qinghaiensis* was the main disseminator of *T. ovis* on the QTP, and this may explain the high infection rate of this pathogen in Tibetan sheep in the present study. The *T. ovis* sequences obtained in the present study were also in the same clade as the *T. ovis* sequence detected in *Rhipicephalus turanicus* from Xinjiang [22], a neighboring province of Qinghai.

The results of this study show that Guoluo and Yushu are significantly impacted by the prevalence of *B. bovis* (*χ*^2^ = 4.56, *df* = 1, *P* = 0.0328) in yaks and the prevalence of *A. ovis* (*χ*^2^ = 23.77, *df* = 1, *P* < 0.0001), *A. bovis* (*χ*^2^ = 52.14, *df* = 1, *P* < 0.0001), *A. capra* (*χ*^2^ = 46.81, *df* = 1, *P* < 0.0001) and *T. ovis* (*χ*^2^ = 50.75, *df* = 1, *P* < 0.0001) in Tibetan sheep. These results may be related to the vegetation type, climate and landform of the two sampling areas. Altitude was shown to have a significant impact on the prevalence of *B. bovis* (*χ*^2^ = 5.77, *df* = 1, *P* = 0.0163) and *T. ovis* (*χ*^2^ = 6.02, *df* = 1, *P* = 0.0141) in yaks, and that of *A. phagocytophilum* (*χ*^2^ = 23.10, *df* = 1, *P* < 0.0001) in Tibetan sheep. Han et al. [56] investigated mixed infections of *Anaplasma* species in ixodid ticks and sheep, and found high co-infections in the latter. Several *Anaplasma* species have been detected in *H. qinghaiensis* [57], which implies that this common tick vector may be responsible for mixed infections with these pathogens.

Previous studies detected *A. marginale* and *B. bigemina* in sheep and yaks in Xinjiang Province, respectively, and *B. motasi*-like L/N/T in sheep in Qinghai Province [9, 12, 18]. However, none of these pathogens, or *B. ovis*, were detected in any of the animals in the present study, which may be due to differences in species distributions and abundances of tick vectors between the sampling sites. The fact that none of these four pathogens were detected in this study also suggests that they may have low prevalences in Guoluo and Yushu or that they may not be present at all.

## Conclusions

This study reports the prevalence of *Anaplasma* spp., *Babesia* spp. and *Theileria* spp. in yaks and Tibetan sheep in Qinghai Province, China. The results of this study add to existing epidemiological information on tick-borne diseases in yaks and Tibetan sheep in Sanjiangyuan, and provide basic data for the development of programs for the prevention and control of TBPs in domestic animals in this area of the QTP hinterland. However, further studies are needed to investigate the relationship between ticks and pathogens in Qinghai Province to provide more information on the epidemiology of TBPs in this area of China.

## Supplementary Information

**Additional file 1: Table S1.** Samples collected from yaks and Tibetan sheep on the Qinghai-Tibetan Plateau (QTP). **Table S2.** Primers used in this study to detect tick-borne pathogens infections in yaks and Tibetan sheep on the QTP.

## Data Availability

The datasets generated or analyzed during the current study are available from the corresponding author on reasonable request. All the nucleotide sequences obtained in this study have been deposited in GenBank and the accession numbers are provided in Table 4.

## Abbreviations

BLASTn: Basic Local Alignment Search Tool nucleotide
PCR: Polymerase chain reaction
QTP: Qinghai-Tibet Plateau
16S rRNA: 16S ribosomal RNA
18S rRNA: 18S ribosomal RNA
TBPs: Tick-borne pathogens
