# Supplementary material for: Molecular detection of Anaplasma spp., Babesia spp. and Theileria spp. in yaks (Bos grunniens) and Tibetan sheep (Ovis aries) on the Qinghai-Tibetan Plateau, China
Source: Parasit Vectors. 2021 Dec 23;14:613. doi: 10.1186/s13071-021-05109-2 (PMC8697493; doi:10.1186/s13071-021-05109-2)
Supplement: Supplementary file 1 — Additional file 1: Table S1. Samples collected from yaks and Tibetan sheep on the Qinghai-Tibetan Plateau (QTP). Table S2. Primers used in this study to detect tick-borne pathogens infections in yaks and Tibetan sheep on the QTP. [file 13071_2021_5109_MOESM1_ESM.docx]

Additional file 1: Table S1. Sample collection of yaks, Tibetan sheep on the Qinghai-Tibetan Plateau (QTP)

| Animal | No.of samples | | | | | | | | | | | | |
| --- | --- | --- | --- | --- | --- | --- | --- | --- | --- | --- | --- | --- | --- |
|  | Guoluo | | | |  | Yushu | | | | | | | Total |
|  | Maqin | Darlag | Banma | Total | | Qumalai | Zhiduo | Chengduo | Yushu | Zaduo | Nangqian | Total |  |
| Yak | 97 | 35 | 78 | 210 | | 22 | 30 | 29 | 53 | 33 | 48 | 215 | 425 |
| Tibetan sheep | 132 | 51 | 0 | 183 | | 50 | 0 | 0 | 0 | 25 | 51 | 126 | 309 |
| Total | 229 | 86 | 78 | 393 | | 72 | 30 | 29 | 53 | 58 | 99 | 341 | 734 |

Additional file 1: Ttable S2. Primers used in this study to detect tick-borne pathogens infections in yaks and Tibetan sheep on the QTP

| Pathogen | Target gene | primers (5'→3') | Fragment (bp) | Note | Reference |
| --- | --- | --- | --- | --- | --- |
| *A. ovis* | *msp4* | TGAAGGGAGCGGGGTCATGGG | 347 |  | [23] |
|  |  | GAGTAATTGCAGCCAGGCACTCT |  |  |  |
| *A. bovis* | 16S rRNA | TCCTGGCTCAGAACGAACGCTGGCGGC | 1433 | 1^st^ PCR | [24] |
|  |  | AGTCACTGACCCAACCTTAAATGGCTG |  |  |  |
|  |  | CTCGTAGCTTGCTATGAGAAC | 551 | Nested PCR |  |
|  |  | TCTCCCGGACTCCAGTCTG |  |  |  |
| *A. capra* | *gltA* | GCGATTTTAGAGTGYGGAGATTG | 1031 | 1^st^ PCR | [25] |
|  |  | TACAATACCGGAGTAAAAGTCAA |  |  |  |
|  |  | GGGTTCMTGTCYACTGCTGCGTG | 793 | Nested PCR |  |
|  |  | TTGGATCGTARTTCTTGTAGACC |  |  |  |
| *A. phagocytophilum* | 16S rRNA | CACATGCAAGTCGAACGGATTATTC | 932 | 1^st^ PCR | [26] |
|  |  | TTCCGTTAAGAAGGATCTAATCTCC |  |  |  |
|  |  | AACGGATTATTCTTTATAGCTTGCT | 546/565 | Nested PCR |  |
|  |  | GGCAGTATTAAAAGCAGCTCCAGG |  |  |  |
| *A. marginale* | *msp4* | CTGAAGGGGGAGTAATGGG | 344 |  | [23] |
|  |  | GGTAATAGCTGCCAGAGATTCC |  |  |  |
| *B. ovis* | 18S rRNA | TGGGCAGGACCTTGGTTCTTCT | 549 |  | [27] |
|  |  | CCGCGTAGCGCCGGCTAAATA |  |  |  |
| *B. bovis* | *SBP4* | AGTTGTTGGAGGAGGCTAAT | 907 | 1^st^ PCR | [28] |
|  |  | TCCTTCTCGGCGTCCTTTTC |  |  |  |
|  |  | GAAATCCCTGTTCCAGAG | 503 | Nested PCR |  |
|  |  | TCGTTGATAACACTGCAA |  |  |  |
| *B. bigemina* | *rap1a* | GAGTCTGCCAAATCCTTAC | 879 | 1^st^ PCR | [28] |
|  |  | TCCTCTACAGCTGCTTCG |  |  |  |
|  |  | AGCTTGCTTTCACAACTCGCC | 412 | Nested PCR |  |
|  |  | TTGGTGCTTTGACCGACGACAT |  |  |  |
| *B. motasi*-like Lintan/Ningxian/Tianzhu | *rap1b* | TGCGCCTTCGAGTTGTACAAGAG | 765 | 1^st^ PCR | [17] |
|  |  | GACGGGTTGCRTAGGCTGAC |  |  |  |
|  |  | TGCGTGGAAGATAGAAAGTTAGCC | 765 | Nested PCR |  |
|  |  | ATGACTGATCTCGACTCTCCATTAGCTGG |  |  |  |
| *Theileria* spp. | 18S rRNA | GAAACGGCTACCACATCT | 778 | 1^st^ PCR | [29] |
|  |  | AGTTTCCCCGTGTTGAGT |  |  |  |
|  |  | TTAAACCTCTTCCAGAGT | 581 | Nested PCR |  |
|  |  | TCAGCCTTGCGACCATAC |  |  |  |
